# High Glucose Environments Interfere with Bone Marrow-Derived Macrophage Inflammatory Mediator Release, the TLR4 Pathway and Glucose Metabolism

**DOI:** 10.1038/s41598-019-47836-8

**Published:** 2019-08-07

**Authors:** Thais Soprani Ayala, Fernando Henrique Galvão Tessaro, Grasielle Pereira Jannuzzi, Leonardo Mendes Bella, Karen Spadari Ferreira, Joilson O. Martins

**Affiliations:** 1 Laboratory of Immunoendocrinology, Department of Clinical and Toxicological Analyses, School of Pharmaceutical Sciences of University Sao Paulo (FCF/USP), São Paulo, Brazil; 2 Laboratory of Cellular Immunology and Biochemistry of Fungus and Protozoa, Department of Pharmaceutical Sciences Analysis, Federal University of São Paulo, São Paulo, Brazil

**Keywords:** Endocrine system and metabolic diseases, Inflammation

## Abstract

Macrophages may be a crucial aspect of diabetic complications associated with the inflammatory response. In this study, we examined how hyperglycaemia, a common aspect of diabetes, modulates bone marrow-derived macrophages (BMDMs) under an inflammatory stimulus. To perform this study, BMDMs from non-diabetic and diabetic (60 mg/kg alloxan, i.v.) male C57BL/6 mice (CEUA/FCF/USP-488) were cultured under normal (5.5 mM) and high glucose (HG, 25 or 40 mM) conditions and stimulated or not stimulated with lipopolysaccharide (LPS, 100 ng/mL). Compared to the BMDMs from the normoglycaemic mice, the LPS-stimulated BMDMs from the diabetic mice presented reduced TLR4 expression on the cell surface, lower phagocytic capacity, and reduced secretion of NO and lactate but greater oxygen consumption and greater phosphorylation of p46 SAPK/JNK, p42 ERK MAPK, pAKT and pPKC-δ. When the BMDMs from the non-diabetic mice were cultured under high-glucose conditions and stimulated with LPS, TLR4 expression was reduced on the cell surface and NO and H_2_O_2_ levels were reduced. In contrast, the diabetic BMDMs cultured under high glucose conditions presented increased levels of lactate and reduced phosphorylation of AKT, PKC-δ and p46 SAPK/JNK but enhanced phosphorylation of the p46 subunit of SAPK/JNK after LPS stimulation. High glucose levels appear to modify macrophage behaviour, affecting different aspects of diabetic and healthy BMDMs under the same LPS stimulus. Thus, hyperglycaemia leaves a glucose legacy, altering the basal steady state of macrophages.

## Introduction

Macrophages play a central role in host defence and are essential to the development of the immune response^1^. In the presence of pathogens, tissue-specific macrophages are activated and begin to secrete immune mediators, playing a crucial role in maintaining the equilibrium of inflammatory signals^2^. An uncontrolled macrophage response promotes host injury and leads to chronic inflammation with loss of tissue homeostasis and function^3^.

The microenvironment that surrounds macrophages influences macrophage behaviour, showing the plasticity of these cells^4–6^. In addition, depending on the external stimulus, macrophages can polarize towards a more pro-inflammatory type of cell, known as M1 macrophages, or an anti-inflammatory cell associated with tissue repair, known as M2 macrophages^7^. M1 macrophages can be generated by stimulation with lipopolysaccharide (LPS), interferon (IFN)-γ, and tumour necrosis factor (TNF)-α, whereas M2-like macrophages are created by stimulation with interleukin (IL)-4, IL-13, and IL-10^4^.

Different stimuli affect metabolic pathways in macrophages, providing evidence of a connection between metabolic and inflammatory processes^8^. Although it macrophage functions and glucose metabolism are known to be associated with each other, the nature of their relationship remains unclear^9^. Diabetes mellitus (DM) is a disorder in which glucose metabolism is impaired that occurs due to a loss of insulin secretion (type 1 diabetes - T1D) and/or a decrease in insulin activity (type 2 diabetes - T2D)^10^. The lack of insulin results in the generation of a hyperglycaemic environment that may be harmful to various systems, organs and cells in the body, including the immune system and macrophages^11,12^.

Recognized by Toll-like receptor-4 (TLR4), LPS triggers different pathways that lead to the production and secretion of inflammatory mediators^13^. The LPS transduction signal is generated by the activation of phosphoinositide 3-kinase (PI3K)/protein kinase B (AKT)^14,15^. The PI3K/AKT pathway may be an important link between the macrophage response induced by TLR4, energy consumption and energetic metabolism^16^. Adenosine 59-monophosphate-activated protein kinase (AMPK) is an energy sensor that acts as an inflammatory and metabolic regulator in most eukaryotic cells^17^. AMPK inactivates mechanistic target of rapamycin (mTOR) components, polarizing macrophages towards a more anti-inflammatory profile. The activation of AKT in this scenario promotes the anti-inflammatory action of AMPK activation^18^. mTOR is integrated into TLR4 signalling, where it activates mechanisms of protein synthesis by activating ribosomal protein S6^19^. Together with MAPK, a group of inflammatory proteins that is composed of extracellular signal-regulated protein kinases 1 and 2 (ERK1/2), c-jun NH2-terminal kinases/stress-activated protein kinases (SAPK/JNK) and p38 proteins, and protein kinase C (PKC) is also involved in the TLR4 inflammatory response^20^. The distinct activities of this protein complex involve cell growth and the induction of the secretion of many pro-inflammatory mediators. Modifying any one of the kinases described above may influence the macrophage inflammatory profile^21,22^.

During LPS activation, macrophage uptake of glucose increases through glucose transporter (GLUT)-1 to fulfil the cellular energy demand^9^. Glucose consumption generates energy in the form of adenosine triphosphate (ATP)^23^, but macrophages can focus on a specific part of the glucose metabolic pathway to respond to some types of stimuli, choosing a pathway to generate the mediators that benefit the specific behaviour needed. Along with this intrinsic regulation, the environment can influence whether macrophages skew glucose metabolism towards glycolysis or oxidative phosphorylation (OXPHOS)^8^.

Hyperglycaemia appears to impair the immune response^24–26^ and the clearance of pathogens by macrophages in diabetic subjects^27^. A lack of glucose homeostasis can be an important key to macrophage deregulation in a hyperglycaemic environment under a variety of stimuli^24,25,28^. Due to the high susceptibility to infections and elevated risk of developing complications after surgery in diabetic patients, failures in inflammation resolution contribute to the high rates of morbidity and mortality in diabetic subjects^29,30^.

In this study, we investigated the disruptions in signalling pathways and cytokine secretion in LPS-stimulated bone marrow-derived macrophages (BMDMs) generated in a high glucose (HG) environment *in vitro* or *in vivo*. The goal of this study was to elucidate the mechanism by which glucose modifies the macrophage profile in the diabetic milieu, which leads to diabetic patients having a high risk of infection events.

## Results

### Alloxan T1D model establishment

We used an alloxan T1D model to obtain macrophages from a hyperglycaemic environment^31,32^. After 10 days, compared to those injected with saline, mice (n = 12) injected with alloxan showed hyperglycaemia (before alloxan injection: 172 ± 12 mg/dL; 10 days after: 574 ± 36 mg/dL; p < 0.05) and loss of body weight (before alloxan injection: 28.3 ± 1.0 g; 10 days after: 25.4 ± 1.0 n = 12 p < 0.05).

### Hyperglycaemia promotes modifications in the macrophage phenotype

**Figure 1:**
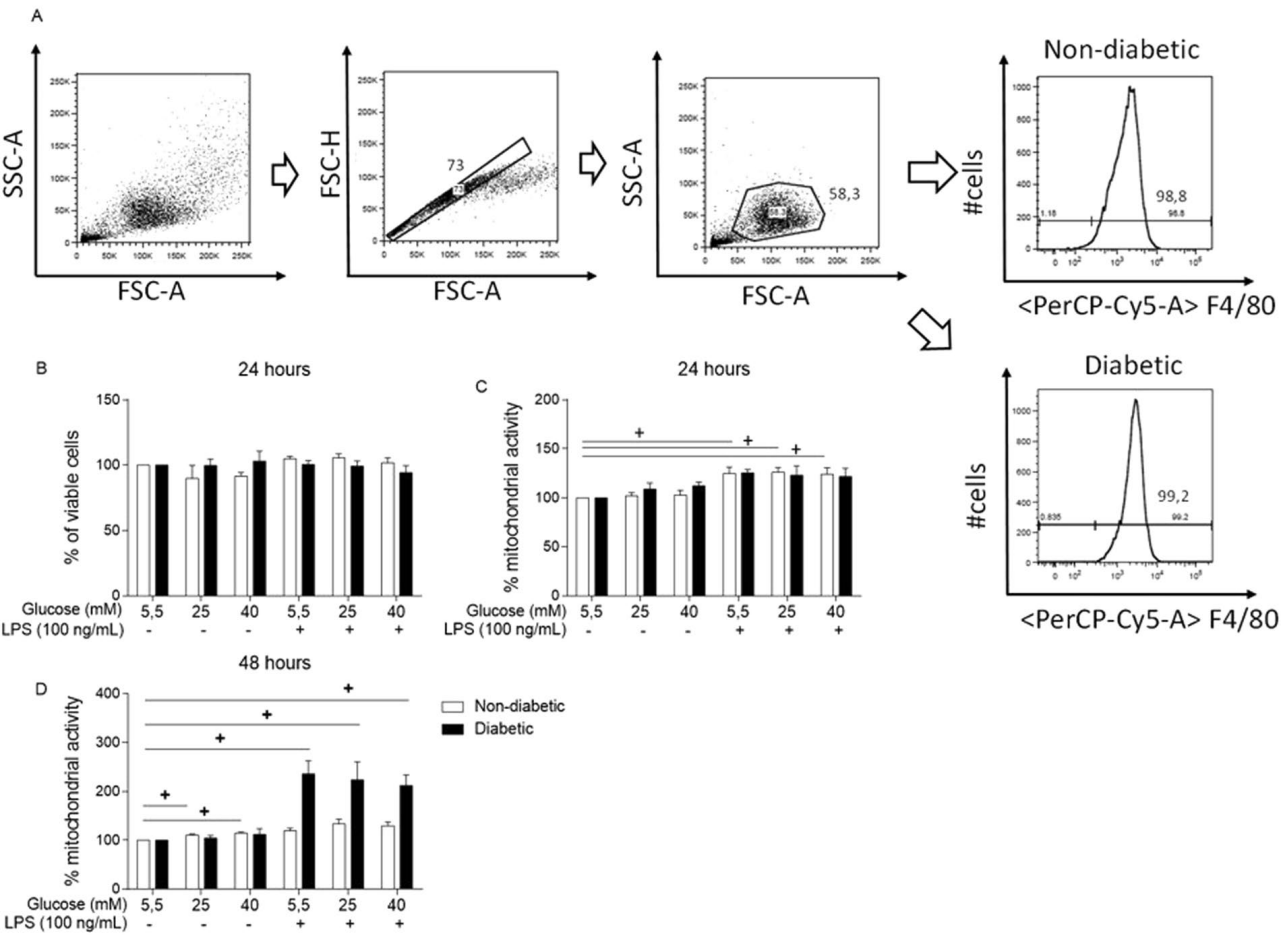
BMDM viability is not modified by hyperglycaemia. (**A**) Gating strategy and F4/80 expression of BMDMs differentiated for 7 days. Viability evaluation by (**B**) PI staining after 24 hours of incubation and by MTT assay after (**C**) 24 hours and (**D**) 48 hours of incubation. *p < 0.05 compared to normoglycaemic BMDMs, ^+^<0.05 compared with control glucose medium. Data are presented as the means ± SEM (N of at least 3).

First, we observed that 95 ± 2% of the viable macrophages differentiated from bone marrow content as described in the Material and Methods section were F4/80+, showing successful differentiation into BMDMs (Fig. 1A). We used propidium iodide (PI) staining followed by flow cytometry to evaluate whether a high glucose medium would affect BMDM viability (Fig. 1B). BMDMs from both groups were cultured under normal glucose (NG, 5.5 mM) or high glucose conditions (25 or 40 mM) with or without LPS for 24 hours. High glucose alone or together with LPS did not alter BMDM viability after 24 hours.

To complement the PI viability assay, we performed the 3-[4,5-dimethylthiazol-2-yl]-2,5 diphenyl tetrazolium bromide (MTT) assay (Fig. 1C,D), as it evaluates mitochondrial activity. The MTT assay is an indirect assay that measures cell viability due to the ability of macrophages to reduce MTT into formazan crystals when the cells are alive^33^. In this context, we compared the high glucose-cultured and LPS-treated BMDMs with the BMDMs cultured in normal glucose (NG, 5.5 mM) medium. LPS stimulation alone promoted an increase in formazan crystal synthesis in the BMDMs from the non-diabetic mice after 24 hours of incubation (Fig. 1C) and in the BMDMs from the hyperglycaemic mice after 48 hours of incubation (Fig. 1D).

**Figure 2:**
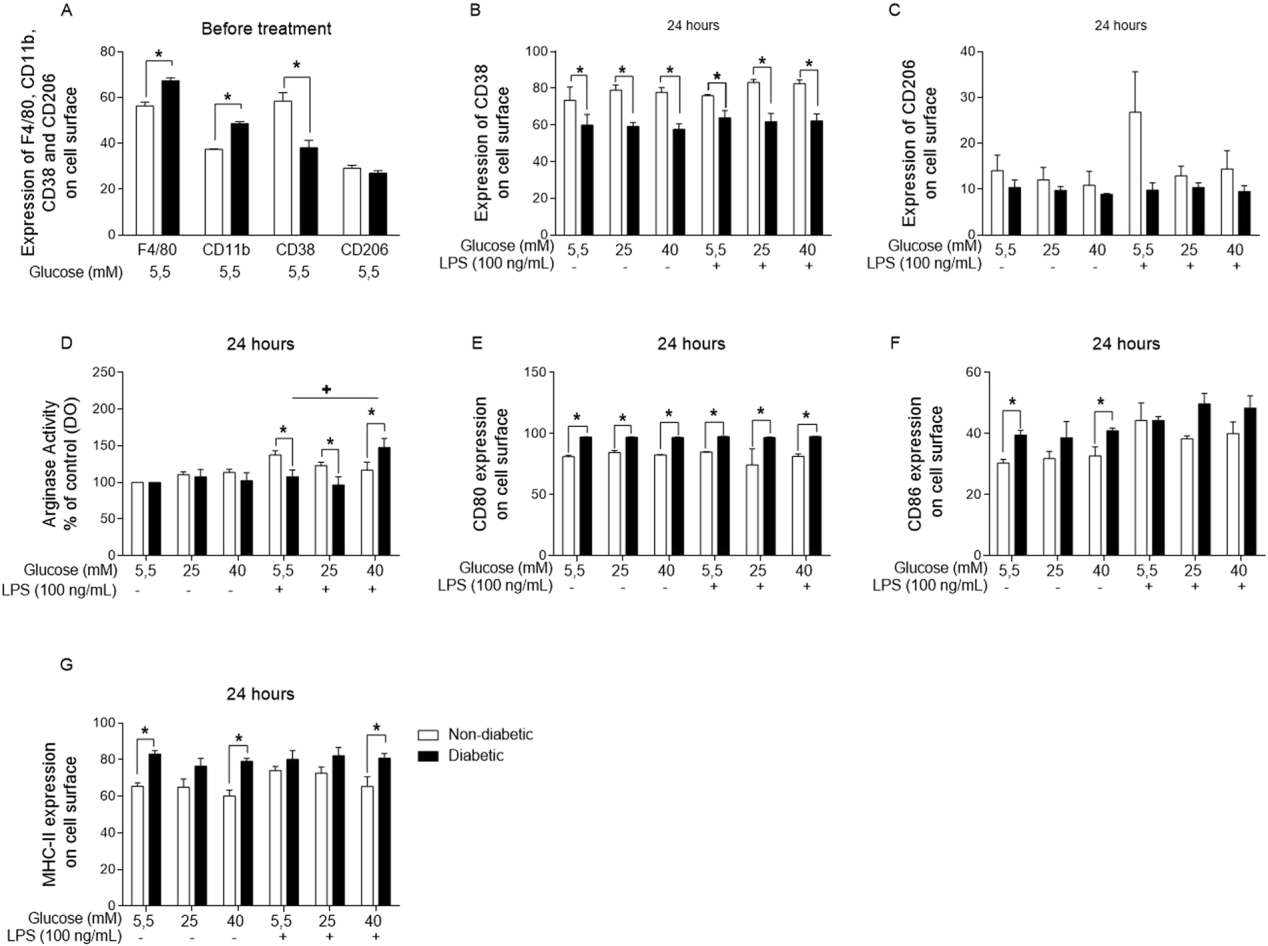
Hyperglycaemia promotes changes in BMDM phenotypes. (**A**) Expression of F4/80, CD11b, CD38 and CD206 before 24 hours of treatment and (**B**) CD38 and (**C**) CD206 expression, (**D**) arginase activity, and (**E**) CD80, (**F**) CD86 and (**G**) MHC-II expression after 24 hours of incubation in normal (5.5 mM) or high glucose (25 or 40 mM) with or without LPS. *p < 0.05 compared to normoglycaemic BMDMs, ^+^<0.05 compared with control glucose medium. Data are presented as the means ± SEM (N = at least 3).

Subsequently, we investigated the phenotype of BMDMs from the non-diabetic and diabetic mice by evaluating BMDM surface markers. The BMDMs from the diabetic mice expressed higher levels of CD11b and F4/80 (Fig. 2A) than those from the non-diabetic mice before any type of treatment. However, after 24 hours of incubation, the expression of CD11b and F4/80 was lower in BMDMs from the diabetic animals than in those from the non-diabetic BMDMs. Following this assay, we investigated whether the level of M1 or M2 markers was different in the BMDMs from the non-diabetic and diabetic mice. Similar to the surface marker expression results, the BMDMs from the diabetic mice expressed lower levels of CD38 than those from the non-diabetic mice before and after receiving the treatments for 24 hours (Fig. 2A,B). However, similar levels of CD206 expression were observed in the BMDMs from the diabetic and non-diabetic mice at both time points (Fig. 2A,C).

Arginase activity is linked to M2 polarization, and our investigation showed that compared with similarly treated BMDMs from the non-diabetic animals, the LPS-stimulated BMDMs from the hyperglycaemic animals presented little arginase activity when exposed to LPS under 5.5 and 25 mM glucose and substantial activity when exposed to 40 mM glucose (Fig. 2D).

In addition, after 24 hours of incubation with normal (5.5 mM) and high (25 or 40 mM) glucose with or without LPS, the BMDMs from the diabetic animals expressed higher levels of CD80 (Fig. 2E), CD86 (Fig. 2F), and MHC-II (Fig. 2G) on the cell surface than the non-diabetic BMDMs.

### Hyperglycaemia modifies glucose metabolism in BMDMs

**Figure 3:**
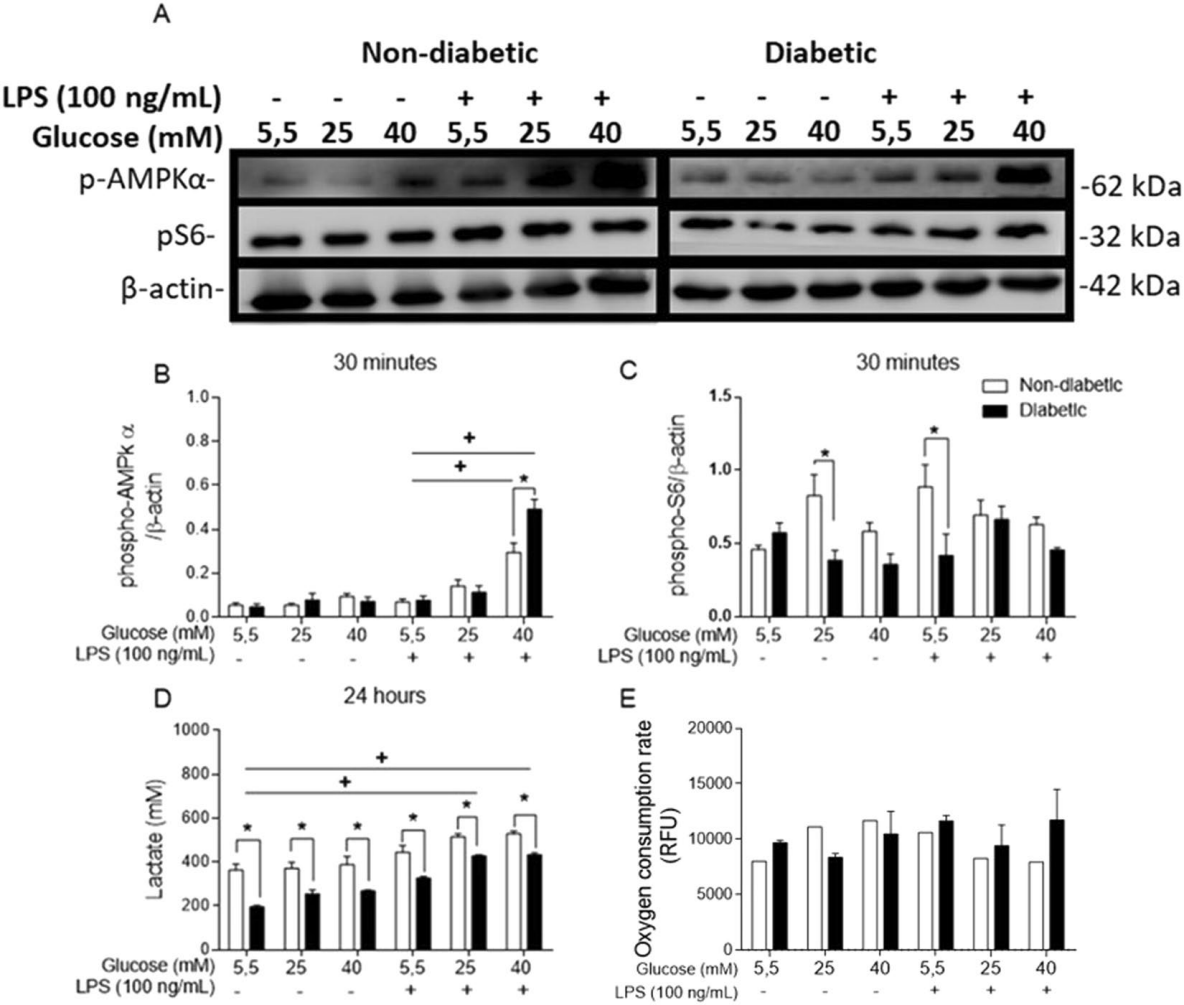
Hyperglycaemia alters the energy metabolism of BMDMs. (**A**) Western blot membranes probed with anti-mouse AMPK, anti-mouse pS6 and anti-mouse β-actin primary antibodies. (**B)** Phospho-AMPK/β-actin ratio. (**C**) Phospho-S6/β-actin ratio. (**D**) Lactate release after 24 hours of incubation. (**E**) Oxygen consumption during the first hour of incubation. *p < 0.05 compared to normoglycaemic BMDMs, ^+^<0.05 compared with control glucose medium. The images of the gels were cropped, with full-length blots/gels presented in Supplementary Fig. S1. The samples were derived from the same experiment, and the gels/blots were processed in parallel. Data are presented as the means ± SEM (N of at least 3).

As an energy sensor, AMPK works to establish both metabolic and energetic homeostasis in cells^8^. Because of its importance in the metabolic and energetic status of macrophages as well as its relationship to inflammatory pathways^34^, AMPK phosphorylation was evaluated by Western blot analysis. AMPKα1 phosphorylation was increased in both the non-diabetic and diabetic BMDMs cultured in 40 mM glucose and stimulated with LPS compared to the BMDMs stimulated with LPS and cultured in 5.5 mM glucose (Fig. 3A,B). Compared to the non-diabetic BMDMs, the diabetic BMDMs stimulated with LPS presented a higher level of activation at 40 mM glucose.

The S6 protein functions downstream of the mTORC1 pathway and is correlated with pro-inflammatory events and metabolic homeostasis. In this study, we observed that after 30 minutes, S6 phosphorylation was highest in the non-diabetic BMDMs maintained in 25 mM glucose and those maintained in 5.5 mM glucose with LPS stimulation (Fig. 3C).

Glycolysis is the first metabolic process in the degradation of glucose into ATP, and anaerobic glycolysis produces lactate as the final product. The non-diabetic BMDMs released more lactate than the BMDMs from the diabetic mice after 24 hours of incubation (Fig. 3D). In addition, LPS increased lactate release in both groups. OXPHOS metabolizes glycolysis products and generates more ATP than glycolysis, and when cells perform OXPHOS, more oxygen (O_2_) is consumed by the mitochondria^35^. Thus, we performed an O_2_ consumption assay during an *in vitro* acute treatment with normal or high glucose with or without LPS. A calculation of the last point of the oxygen consumption subtracted by the first point showed no significant differences between the groups. However, high glucose without LPS appeared to increase oxygen consumption in the non-diabetic BMDM (Fig. 3E).

### Phagocytosis is impaired in BMDMs from diabetic mice

**Figure 4:**
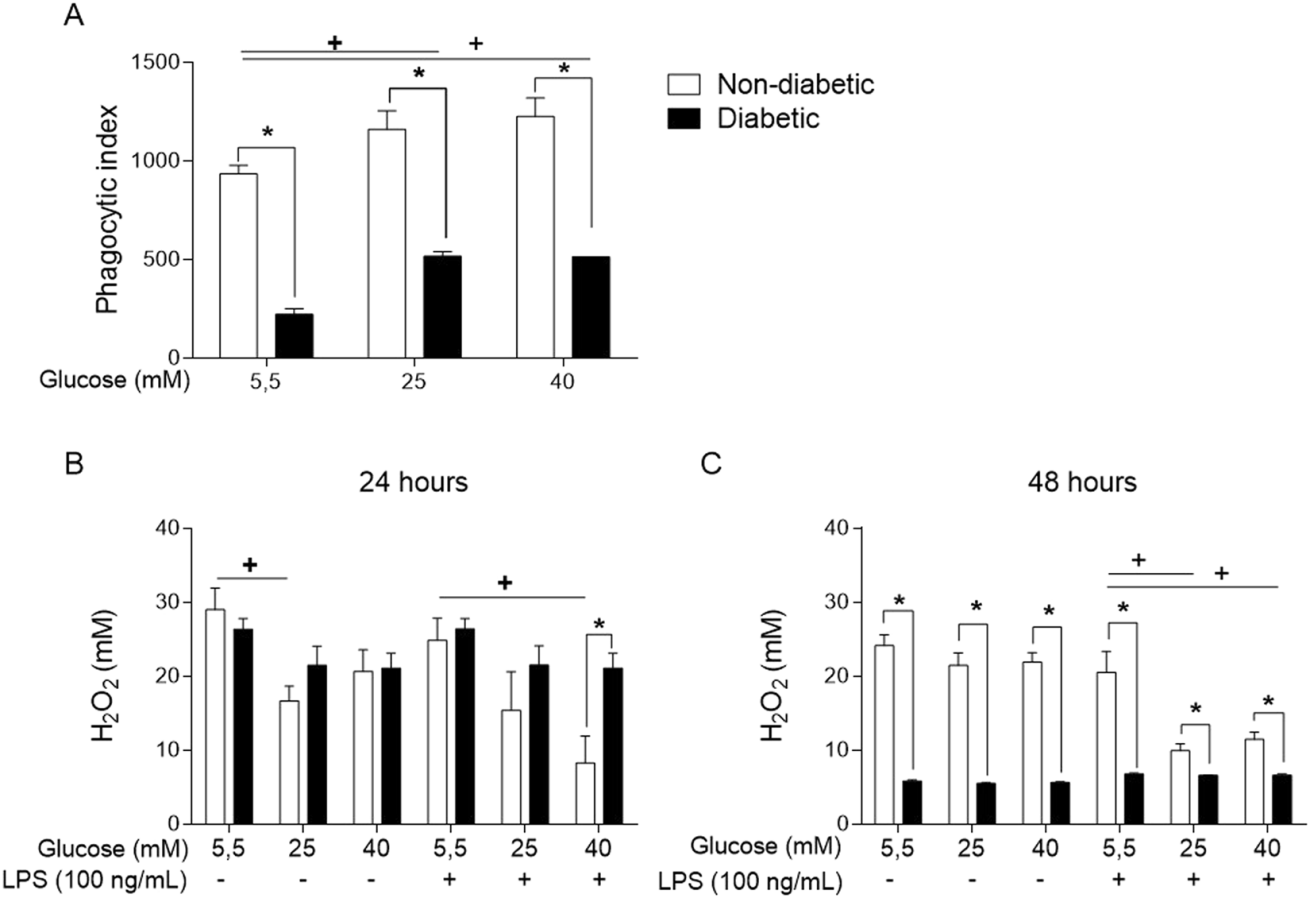
Phagocytosis and reactive hydrogen species production is impaired under hyperglycaemia. (**A**) Phagocytosis of opsonized RBCs. H_2_O_2_ levels in the supernatant of BMDMs cultured in normal (5.5 mM) or high glucose (25 or 40 mM) with or without LPS (100 ng/mL) for (**B**) 24 hours or (**C**) 48 hours. *p < 0.05 compared to normoglycaemic BMDMs, ^+^<0.05 compared with control glucose medium. Data are presented as the means ± SEM (N = 3).

To determine whether high glucose would affect the phagocytic capacity of BMDMs, we used opsonized red blood cells (RBCs) from sheep, and after allowing the BMDMs to interact with the opsonized RBCs on a coverslip, we evaluated the phagocytic index. Phagocytosis was less effective in the diabetic BMDMs than the non-diabetic BMDMs (Fig. 4A). However, when these cells were exposed to high glucose medium *in vitro*, the non-diabetic BMDMs exhibited no changes in phagocytosis, whereas the diabetic BMDMs had higher opsonized RBC intake than the cells maintained in a normal glucose environment.

Macrophages secrete hydrogen peroxide (H_2_O_2_), a potent mediator that is capable of destroying pathogens by lysing their cell membrane. We used an Amplex Red assay (Invitrogen®) to detect this mediator in the supernatant after two short periods, 24 (Fig. 4B) and 48 hours (Fig. 4C). Compared with the non-diabetic BMDMs, the diabetic BMDMs showed obviously impaired H_2_O_2_ release after 48 hours. In addition, high glucose promoted a decrease in H_2_O_2_ release in the non-diabetic BMDMs *in vitro*, with this phenomenon occurring after 24 hours with or without LPS and after 48 hours with LPS.

### Hyperglycaemia decreases TLR4 expression in BMDMs

**Figure 5:**
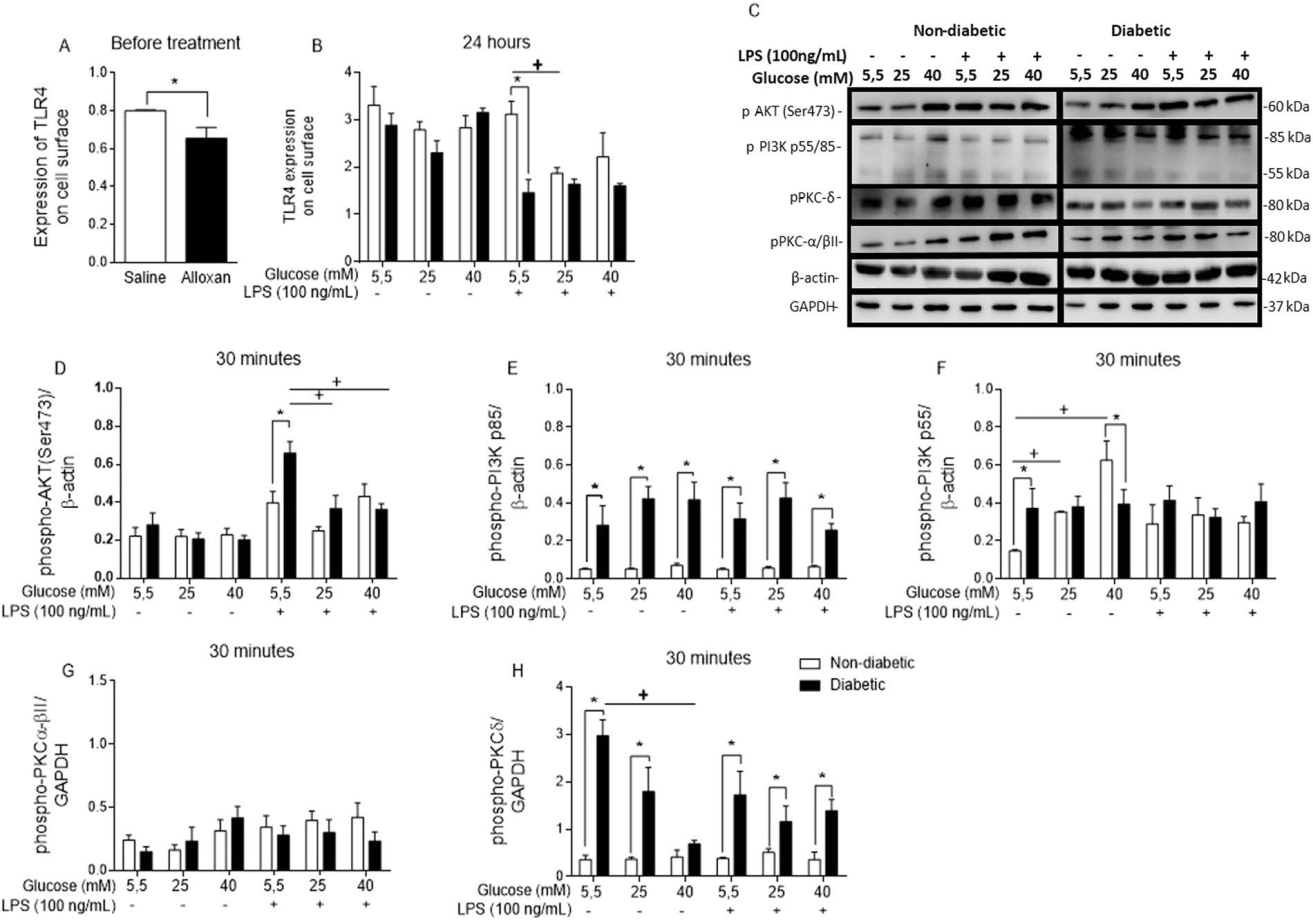
LPS-TLR4 intracellular signalling is disrupted by hyperglycaemia. TLR4 cell surface expression (**A**) before and (**B**) after 24 hours of incubation with different glucose concentrations with or without LPS. (**C**) Western blot membrane probed with anti-phospho-AKT, anti-phospho-PI3K p85/p55, anti-phospho-PKC-α/βII, anti-phospho-PKC-δ, anti-mouse β-actin, and anti-mouse GAPDH primary antibodies. (**D**) Phospho-AKT/β-actin ratio. (**E,F**) Phospho-PI3k p85/p55/β-actin ratios. (**G**) Phospho-PKC-α/βII/GAPDH ratio. (**H**) Phospho-PKC-δ/GAPDH ratio. *p < 0.05 compared to normoglycaemic BMDMs, ^+^<0.05 compared with control glucose medium. The images of the gels were cropped, with full-length blots/gels presented in Supplementary Fig. S2. The samples were derived from the same experiment, and the gels/blots were processed in parallel. Data are presented as the means ± SEM (N of at least 3).

To verify whether a high glucose environment disturbs TLR4 expression, we evaluated TLR4 expression on the surface of BMDMs by flow cytometry before any treatment (Fig. 5A) and after a 24 hour incubation using normal and high glucose with or without LPS stimulation (Fig. 5B). The diabetic BMDMs expressed lower levels of TLR4 at both time points. Additionally, the non-diabetic BMDMs maintained in high glucose (25 mM) medium for 24 hours with LPS stimulation expressed lower levels of TLR4 on the cell surface than the control BMDMs stimulated with LPS.

### High glucose modifies inflammatory signalling pathways activated by LPS

To study TLR4-LPS signalling, we investigated molecules involved in this pathway. High glucose altered AKT activation in the BMDMs from the diabetic mice, as shown by higher phosphorylation of AKT Ser473 in these cells than was observed in the non-diabetic BMDMs. Furthermore, when the diabetic BMDMs were cultured under *in vitro* high glucose (25 mM) and LPS stimulation conditions, a decrease in AKT Ser473 phosphorylation was observed (Fig. 5D).

PI3K p55 subunit phosphorylation in the non-diabetic BMDMs was the highest when these cells were cultured in high glucose (40 mM) medium without LPS (Fig. 5F). However, PI3K p85 phosphorylation was higher in the diabetic BMDMs than in the non-diabetic BMDMs (Fig. 5E).

PKC is a family of proteins that is associated with TLR4 signalling and phagocytosis. We observed that the BMDMs originating from the diabetic mice presented higher levels of PKC-δ phosphorylation than those from non-diabetic mice (Fig. 5H). Nevertheless, high glucose (40 mM) led to minor phosphorylation of this PKC isoform in the BMDMs from the diabetic mice (Fig. 5H). No modifications in PKC-α/βII phosphorylation were observed (Fig. 5F).

### High glucose levels influence the MAPK signalling pathway

**Figure 6:**
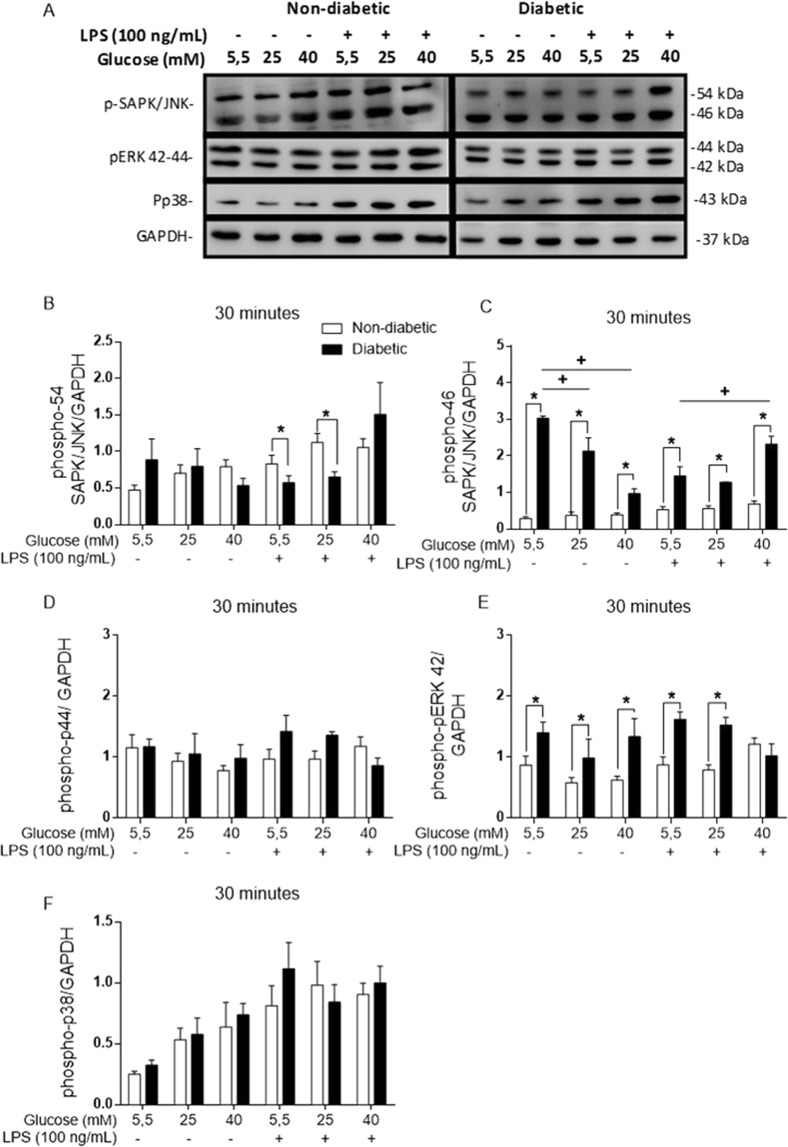
MAPKs are phosphorylated at different levels in diabetic BMDMs. (**A**) Western blot membrane probed with anti-phospho-SAPK/JNK, anti-phospho-42/44, anti-phospho-P38 and anti-GAPDH primary antibodies. (**B**) Phospho-SAPK/JNK p54/GAPDH ratio. (**C**) Phospho-SAPK/JNK p46/GAPDH ratio. (**D**) Phospho-p44/GAPDH ratio. (**E**) Phospho-p42/GAPDH ratio. (**F**) Phospho-P38/GAPDH ratio. *p < 0.05 compared to normoglycaemic BMDMs, ^+^<0.05 compared with control glucose medium. The images of the gels were cropped, with full-length blots/gels presented in Supplementary Fig. S3. The samples were derived from the same experiment, and the gels/blots were processed in parallel. Data are presented as the means ± SEM (N = 4).

MAPKs are essential to the inflammatory response. The diabetic BMDMs showed higher levels of both SAPK/JNK p46 (Fig. 6C) and ERK p42 (Fig. 6E) phosphorylation but lower levels of SAPK/JNK subunit p54 phosphorylation than the non-diabetic BMDMs. Nevertheless, when the BMDMs from the diabetic mice were maintained in a high glucose environment (25 or 40 mM) without LPS stimulation, a decrease in p46 phosphorylation occurred, but when stimulated by LPS, the cells showed an increase in p46 phosphorylation when cultured with 40 mM glucose.

No differences were observed in the p38 phosphorylation levels of the non-diabetic and diabetic BMDMs during exposure to different levels of glucose and LPS (Fig. 6F).

### Short periods of high glucose exposure modifies inflammatory mediator secretion by BMDMs

**Figure 7:**
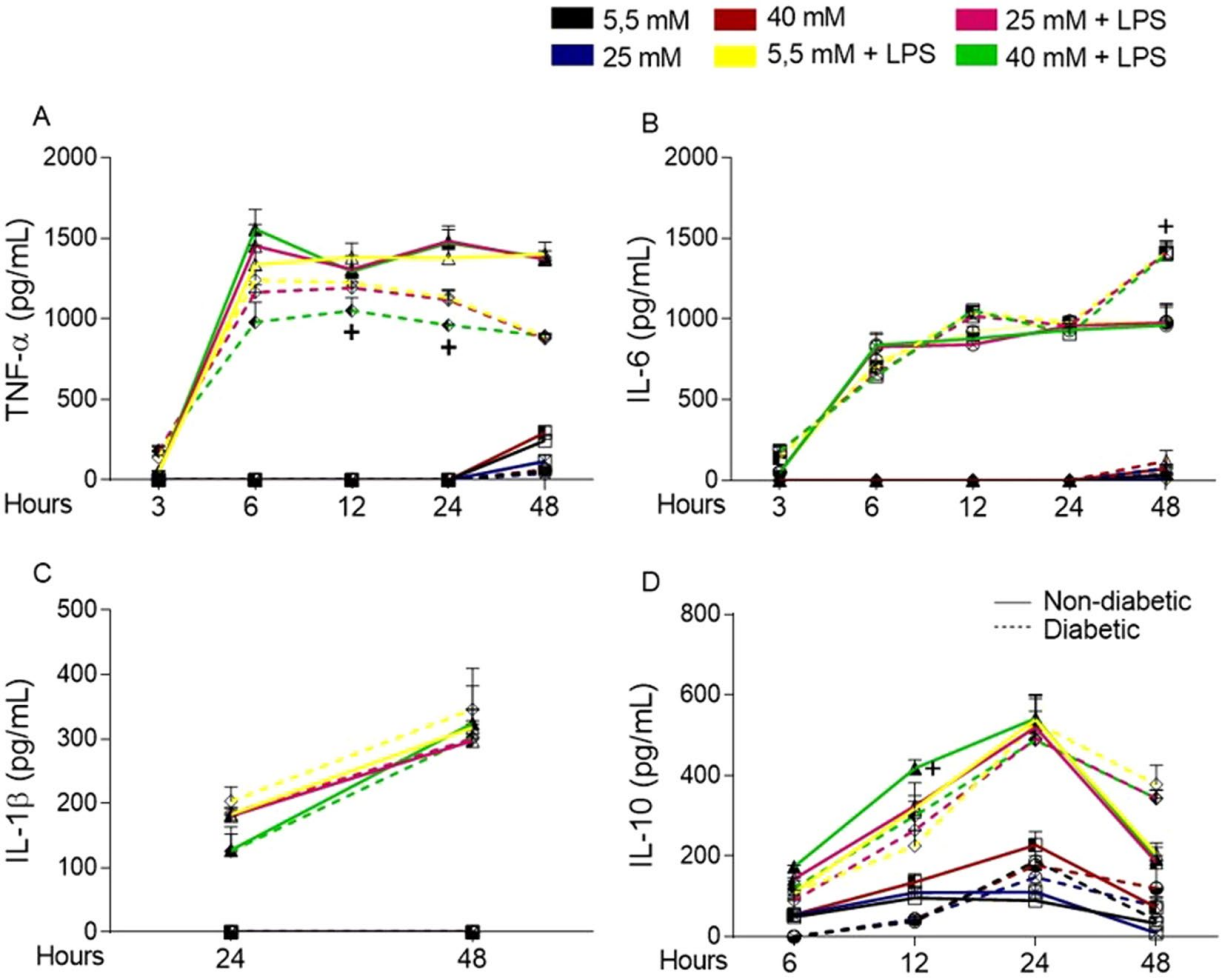
Short-term exposure to high glucose promotes a slight modification in the cytokine release into the supernatant of BMDMs cultured under normal (5.5 mM) or high glucose (25 or 40 mM) conditions with or without LPS (100 ng/mL). (**A**) TNF-α, (**B**) IL-6, (**C**) IL-1β and (**D**) IL-10 levels. *p < 0.05 compared to normoglycaemic BMDMs, ^+^<0.05 compared with control glucose medium. Data are presented as the means ± SEM (N = 6).

Glucose modifies the way that BMDMs respond to LPS in terms of signalling pathways and energy production. In this study, we measured the levels of secreted TNF-α (Fig. 7A), IL-6 (Fig. 7B), IL-1β (Fig. 7C), and IL-10 (Fig. 7D). When the BMDMs were cultured with 5.5, 25, or 40 mM glucose, we detected TNF-α and IL-6 in the supernatant of BMDMs only after 48 hours of incubation, and IL-10 release was detected at 3 hours in the non-diabetic BMDM supernatant. For diabetic BMDMs, the cytokine levels were quantified at 12 hours. Indeed, the non-diabetic BMDMs secreted higher levels of both TNF-α and IL-10, although IL-6 secretion was lower in the non-diabetic BMDM culture than the diabetic BMDM culture.

Given the effect of high glucose *in vitro*, after 48 hours in 25 mM glucose, the BMDMs from the non-diabetic mice released less TNF-α than the BMDMs cultured in 5.5 mM glucose. High glucose also affected IL-10 release in the same group of BMDMs, leading to higher levels of this cytokine under high glucose conditions than under 5.5 mM glucose conditions after 6 hours of incubation. In addition, the BMDMs from the diabetic mice released lower levels of TNF-α after 12 and 24 hours.

### Long-term high glucose exposure modifies inflammatory mediator secretion by BMDMs

The BMDMs were also incubated for 7 days, with fresh medium added to the cells on day 4. This long-term exposure to high glucose promoted different modifications in a wide range of inflammatory mediators.

**Figure 8:**
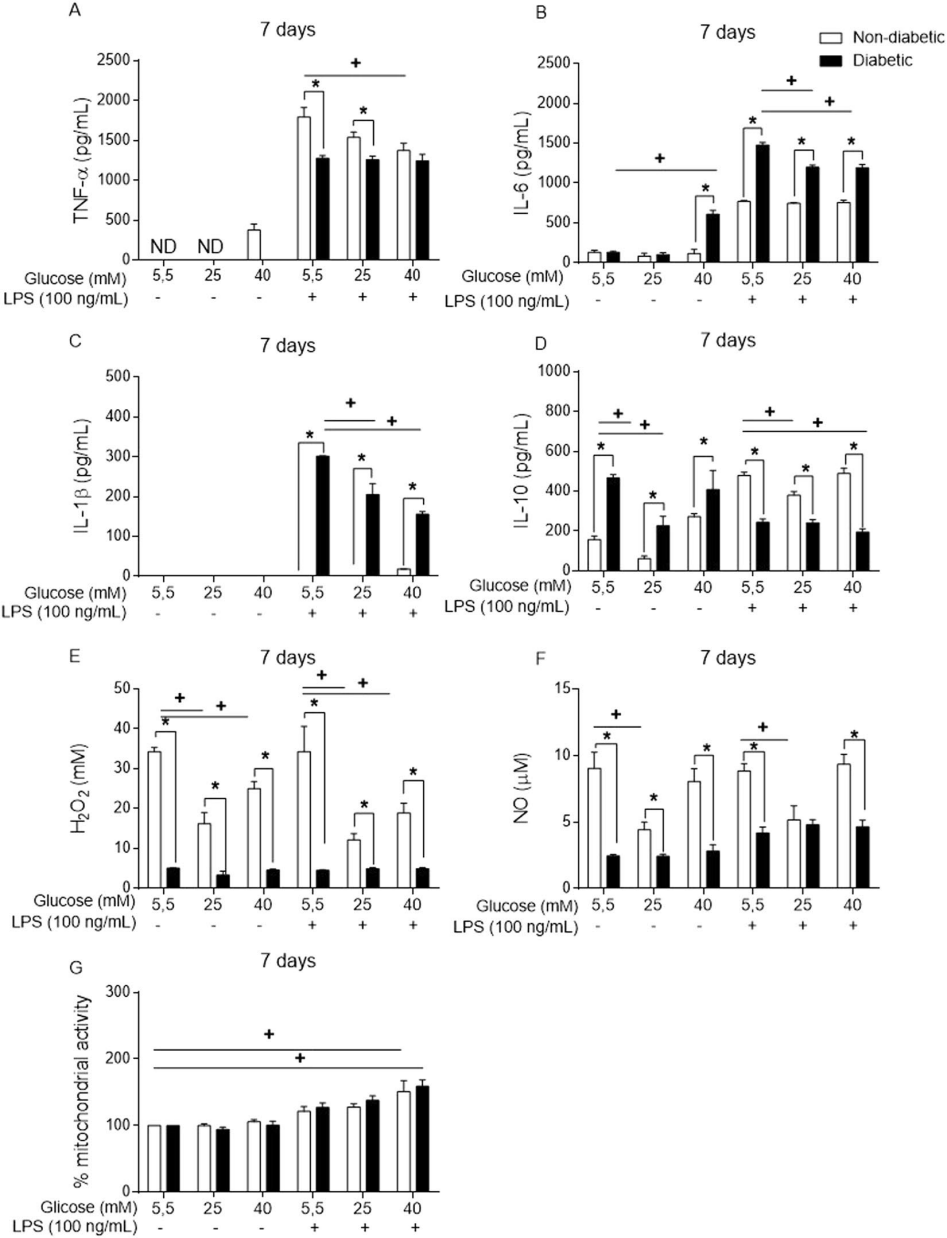
Long-term exposure to high glucose levels promotes the modification of inflammatory mediator release into the supernatant of BMDMs. (**A**) TNF-α, (**B**) IL-6, (**C**) IL-1β, (**D**) IL-10, (**E**) H_2_O_2_ and (**F**) NO levels. (**G**) Viability measured by MTT assay after 7 days of incubation. *p < 0.05 compared to normoglycaemic BMDMs, ^+^<0.05 compared with control glucose medium. Data are presented as the means ± SEM (N = 6).

The non-diabetic BMDMs in a high glucose environment released more TNF-α (40 mM) (Fig. 8A) and less IL-10 (25 mM) than the non-diabetic BMDMs in a normal glucose environment (Fig. 8D). When stimulated with LPS, compared to those cultured in normal glucose medium, the BMDMs cultured in high glucose exhibited decreases in TNF-α (Fig. 8A) and IL-10 (Fig. 8D) secretion. In contrast, the levels of IL-1β and IL-6 remained unchanged in the non-diabetic BMDMs. Thus, in a high glucose environment under LPS stimulus, the diabetic BMDMs secreted low levels of IL-6 (25 and 40 mM) (Fig. 8B) and IL-1β (25 and 40 mM) (Fig. 8C), while also secreting levels of IL-10 under high glucose (25 mM) (Fig. 8D).

The release of H_2_O_2_ and nitric oxide (NO) from BMDMs was also influenced by long-term high glucose exposure. Compared to that observed for the non-diabetic BMDMs, the level of H_2_O_2_ (Fig. 8E) and NO (Fig. 8F) release from the diabetic BMDMs was lower. In this context, high glucose promoted a decrease in H_2_O_2_ (Fig. 8E) and NO (Fig. 8F) secretion from the non-diabetic BMDMs.

The MTT assay with the long-term exposed BMDMs also showed no decrease in the mitochondrial activity of the BMDMs cultured under different glucose concentrations and LPS for 7 days (Fig. 8G).

## Discussion

The primary triggering factor for complications in T1D is hyperglycaemia^36^. Diabetes impairs glucose control throughout the entire body, with many cells failing to internalize and metabolize glucose due to the lack of insulin, while other types of cells remain in constant contact with high glucose concentrations, promoting intracellular hyperglycaemia^37^.

BMDMs are the precursors of macrophages that are recruited to sites of inflammation^38^, and these cells are different from tissue-specific macrophages^39^. Diabetes has been demonstrated to have a huge impact on haematopoiesis^40,41^, and most of the literature consistently shows that diabetes greatly impairs the generation of monocytes^42–45^. Haematopoiesis is a complex process that involves a large number of growth factors, cytokines and cells to be successful. To minimize these effects, our study was conducted using the same protocol to differentiate bone marrow cells from diabetic and non-diabetic mice^38,46^. Our hypothesis was that the differentiation of macrophages under normal glucose conditions would elucidate aspects of glycaemia memory, especially those originating from diabetic bone marrow. In this context, we assessed whether glucose memory would remain in macrophages even though the bone marrow cells differentiate under normal glucose conditions.

Initially, neither diabetes nor the different glucose concentrations used *in vitro* altered cell viability. The MTT assay measures succinate dehydrogenase activity and it is also used to evaluate cell viability, as viable cells convert MTT into formazan crystals^33,47^. Our results showed that LPS promoted an increase in MTT activity after 24 hours in the non-diabetic BMDMs and after 48 hours in the diabetic BMDMs. In addition, LPS is known to promote an increase in mitochondrial activity, and in this assay it appeared that the diabetic BMDMs exhibited a delay in this increase.

Macrophage polarization is described as the plastic capacity of these cells to behave as anti- or pro-inflammatory mediators according to the microenvironment^7^. We observed that the BMDMs from the diabetic mice had lower levels of the M1 marker CD38, whereas the levels of CD206, an M2 marker, remained unaltered. Arginase activity is considered to be an M2 marker, and we observed that arginase activity was lower in the diabetic BMDMs than the non-diabetic BMDMs. Taken together, the results of the M1 and M2 markers analysis did not elucidate a polarization profile that could easily define diabetic BMDM polarization as a specific phenotype. Because of their plasticity, the capacity of BMDMs to induce inflammation or damage resolution can explain the lack of a clear profile when comparing diabetic and non-diabetic BMDMs. In contrast, Wang *et al*.^48^ showed that a cell lineage of macrophages, RAW 264.7 cells, presented an M2-like macrophage phenotype when cultured under high glucose conditions^48^. Sun *et al*.^49^ also showed an increase in arginase activity when peritoneal macrophages were cultured under high glucose conditions *in vitro*^49^.

Molecules such as CD80, CD86 and MHC-II participate in the activation of the adaptive immune response^50^. In this context, we verified that after 24 hours, the BMDMs from the diabetic animals presented higher levels of these three markers than the non-diabetic BMDMs. This difference showed how hyperglycaemia promotes changes in the macrophage phenotype *in vivo*.

Glucose metabolism has an important role in macrophage activation^51^. The TLR4-LPS signalling pathway triggers metabolic changes in macrophages. As an energy sensor that links nutrient availability to inflammatory and metabolic pathways, mTORC1 uses S6K as a downstream protein to induce protein synthesis^52^. In this study, phosphorylation of the S6 protein was observed to be higher in the non-diabetic BMDMs (25 or 5.5 mM glucose + LPS) than in diabetic BMDMs. Cytokines that are expressed in response to LPS, such as IL-6 and IL-10, are also regulated by the mTOR cascade^53^. In addition, AMPKα1 is a protein that is also involved in energy and metabolic regulation, and when phosphorylated, AMPK regulates aspects of the anti-inflammatory pathway, including the inhibition of the mTOR cascade, which decreases inflammatory protein synthesis^34^. In this study, we observed that high glucose appears to activate AMPKα1 phosphorylation both *in vivo* and *in vitro*. LPS is known to promote an increase in glucose consumption, which would subsequently inhibit AMPKα1 due to AMPKα1 driving a catabolic pathway that obstructs ATP production. Thus, when activated, AMPKα1 promotes the retention of ATP^8^.

Glycolysis is the pathway that is responsible for glucose metabolism and energy generation in the form of ATP, with lactate being converted to pyruvate as the final product. LPS drives a higher energy demand in cells, promoting glycolysis to supply the energy needed to secrete a range of immune mediators^54^. According to our results, the diabetic BMDMs produced less lactate, while high glucose appeared to increase O_2_ consumption by the non-diabetic BMDMs, indicating that hyperglycaemia affected the efficiency of glucose usage and promoted an inflammatory response in the cells. As glycolysis is crucial for immune cell function^55^, impaired lactate release may be an indication of enhanced OXPHOS^56^ or glucose metabolism via a different route. Importantly, changes in glucose metabolism in macrophages can modify the cell response to pathogenic stimuli.

As TLR4 sensing is the primary means by which LPS is recognized, TLR4 activation and signalling must be tightly controlled^57^. In this study, we observed that high glucose downregulates TLR4 expression *in vivo* and *in vitro* and impairs its return to the BMDM surface, and this change may bias pathogen recognition by BMDMs, interfering with antigen clearance.

The PI3K/AKT pathway is triggered by TLR4 activation and is involved in several cell processes that sustain the inflammatory responses initiated by macrophage LPS activation^58^. Under high glucose conditions, the non-diabetic BMDMs exhibited an increase in the phosphorylation of the PI3K subunit p85 isoform p55 without LPS stimulation. PI3K has many subunits^59^. PI3K p85 is a regulatory subunit, and macrophages deficient in this subunit tend to secrete more TNF-α and IL-6 upon LPS stimulation^58^.

AKT is the primary substrate of PI3K and is associated with the regulation of inflammatory and energetic metabolic responses^52^. Chronic glucose excess activates mTORC1 in an exacerbated manner that promotes AKT inhibitory feedback, and this inhibition can influence macrophage polarization^52^. Correspondingly, LPS stimulation of BMDMs from the diabetic animals led to higher AKT phosphorylation than stimulation of the non-diabetic BMDMs when maintained in 5.5 mM glucose. However, when the same BMDMs from the diabetic animals were maintained in 25 mM glucose plus LPS, a decrease in AKT phosphorylation occurred, which may indicate that a high glucose concentration alters AKT phosphorylation for both the non-diabetic and diabetic BMDMs. Wang *et al*.^48^ demonstrated that high glucose leads to activation of RAW 264.7 cells through the PI3K/AKT signalling pathway, which induces the M2 macrophage phenotype defined by arginase and CD206 expression, both of which are blocked when PI3K is inhibited^48^. Similarly, Nandy *et al*.^60^ showed an increase in AKT phosphorylation in THP-1 cells exposed to a high glucose medium^60^. Thus, the PI3K/AKT likely plays an important role in macrophage behaviour in hyperglycaemia.

In the MAPK family, ERK1/2, p38 MAPK, and SAPK/JNK mediate the activation of AP1 and regulate the inflammatory response^61^. Our results showed that the hyperglycaemic BMDMs had more phosphorylated SAPK/JNK p46 and ERK p44 than the non-diabetic BMDMs, although no changes in p38 phosphorylation were observed. Interestingly, high glucose led to a decrease in SAPK/JNK p46 phosphorylation in diabetic BMDMs *in vitro*, but when these cells were stimulated with LPS, SAPK/JNK p46 phosphorylation increased under high glucose conditions. Although the SAPK/JNK subunits p46 and p54 share the same substrates, they may have importantly different and distinct functions^49^.

ERK1/2 plays a role in promoting the proliferation and synthesis of extracellular matrix^62^. Sun *et al*. showed that ERK1/2 is involved in M2 macrophage polarization in RAW 264.7 cells, primarily when these cells were stimulated with TGF-β in a high glucose environment. ERK 1/2, also called ERK 42/44, are homologous isoforms that share the same substrate and are important for keeping cells alive and responsive^63^. Despite modifications in the ERK p42 phosphorylation levels, the BMDMs did not exhibit changes in their viability.

The increased phosphorylation of MAPK proteins in diabetic BMDMs under different levels of glucose stimulation could lead to strong AP1 and NF-κB translocation to the nucleus with substantial inflammatory mediator release. However, the BMDMs from diabetic mice secreted less TNF-α and IL-10 but more IL-6 than the non-diabetic BMDMs. Thus, although the inflammatory pathway appears to be activated under hyperglycaemic conditions, the result of this activation does not lead to efficient mediator release.

Together with TLR4-LPS pathway modification, hyperglycaemia did alter the secretion of some inflammatory mediators. Cytokines such as TNF-α, IL-6, IL-1β and IL-10 as well as reactive oxygen species (ROS) and NO are important mediators released by macrophages to eliminate invading bacteria^39^. In our assays, the BMDMs from the diabetic mice secreted more IL-6 and IL-1β but less TNF-α, IL-10, H_2_O_2_ and NO than the non-diabetic BMDMs.

Previous studies have shown that high glucose modifies the release of cytokines such as TNF-α^49,64,65^, IL-6^66^, IL-1β^67^ and IL-10^68^ by macrophages. Altogether, these cytokines play key roles in orchestrating inflammation, and disrupting this mechanism promotes an imbalance in the microenvironment. In our results, anti-inflammatory feedback appeared to be impaired, as IL-10 release was disrupted and delayed in the diabetic BMDMs. Overall, high glucose altered the secretion of some mediators *in vitro*, with the largest changes occurring in the macrophages with long-term exposure to high glucose.

This difference in behaviour between the BMDMs from the non-diabetic and diabetic mice when stimulated by LPS suggests that hyperglycaemia promotes changes in macrophage precursors *in vivo*. After 10 days in a hyperglycaemic environment and 7 days of differentiation of the bone marrow component under normal glucose conditions, the BMDMs from the diabetic mice still had a different inflammatory profile.

Because these cells originate from bone marrow, it appears that they are compromised but do not simply fit into the M1 or M2 macrophage classification. This result reinforces the hypothesis that in a hyperglycaemic state, macrophage precursors are already compromised when recruited^49^, and this state may be strongly correlated with the high susceptibility of diabetic subjects to infections.

The effects of high glucose on macrophages have been shown to be primarily due to high glucose itself^69–73^. Hyperglycaemia disrupts many cellular functions, and the “legacy effect” triggered by uncontrolled glycaemia may be associated with a short or long period of high glucose exposure^74^. It is possible that diabetic BMDMs cannot overcome high glucose to maintain regular inflammatory functions, promoting the establishment of “glycaemic memory”^74^. In addition, it appears that non-diabetic BMDMs are more resistant to changes triggered by persistent high glucose than diabetic BMDMs, and a long exposure time is necessary to promote substantial changes in the levels of cytokine release.

## Methods

### Animals

In this study, we used 9–11-week-old C57BL/6 mice that weighed approximately 25–30 g. The mice were maintained at 23 ± 2 °C under a 12 hour light/dark cycle and were provided food and water ad libitum. For the T1D model, 60 mg/kg of alloxan (Sigma-Aldrich®) was administered intravenously, with saline administered using the same approach for non-diabetic animals. All of the animals were weighed and had blood glucose measured before and 10 days after the injection. Our study was approved by the Ethics Committee on Animal Use (CEUA) at the School of Pharmaceutical Sciences (FCF), the University of São Paulo, Brazil (protocol number: CEUA/FCF/488), and all procedures were performed in strict accordance with the principles and guidelines of the National Council for the Control of Animal Experimentation (CONCEA).

### BMDMs and cell culture

To obtain BMDMs, animals were euthanized with ketamine (270 mg/kg) and xylazine (30 m/kg). The bone marrow content was collected from non-diabetic and diabetic animals after 10 days of saline or alloxan injection, respectively^32^. The cells were obtained by flushing the inside of the femurs with sterile PBS. To differentiate the bone marrow into BMDMs, the bone marrow was treated with a mixture of 50% RPMI-1640 medium (Gibco® by Life Technologies Thermo Fisher Scientific, Waltham, MA, USA), 30% L929 cell conditioned medium (LCCM) and 20% foetal bovine serum (FBS) (Sigma-Aldrich, St. Louis, MO, USA) for 7 days, with fresh medium added on the 4th day to maintain the cells^38^.

After 7 days, the cells were harvested with cold sterile PBS, counted by dead cell exclusion using trypan blue (Gibco® by Life Technologies, Thermo Fisher Scientific), and seeded in a mixture of RPMI-1640 medium (Gibco® by Life Technologies, Thermo Fisher Scientific), LCCM and FBS (85% RPMI-1640 medium, 5% LCCM and 10% FBS) into different types of plates specific for each of the assays described below. The cells were seeded at a density of 2 × 10^5^ and 2 × 10^6^ for the 96- and 6-well plates, respectively.

After 12 hours of incubation, the supernatant was discarded, and the BMDMs were cultured in RPMI-1640 medium without glucose, with the glucose concentration adjusted using a glucose solution (both from Gibco® by Life Technologies Thermo Fisher Scientific). The cells were cultured in three different glucose concentrations (5.5 mM as control medium and 25 or 40 mM as high glucose medium)^64,75,76^ in the presence or absence of 100 ng/mL LPS (Sigma-Aldrich)^32^.

### MTT assay

To measure cell viability, we assessed mitochondrial activity via an MTT assay. Briefly, cells were seeded in a 96-well culture plate, and after 24 hours, 48 hours or 7 days of incubation with normal or high glucose medium with or without LPS, we added MTT (5 mg/mL) and incubated the cells for another 4 hours. Subsequently, we added dimethyl sulfoxide (DMSO) and read the plate with a microplate reader at 540 nm. The results are presented as a percentage of the control^77^.

### Time course culture

BMDMs were seeded at 2 × 10^6^ per well and cultured under normal glucose (5.5 mM) or high glucose (25 or 40 mM) conditions with or without LPS for different times. Subsequently, the cellular supernatant was collected and used to measure the levels of lactate, cytokines, H_2_O_2_ and NO.

### Lactate measurements

The lactate levels in the cell supernatants were measured after 24 hours of culturing under the conditions described above using an L-lactate assay kit (ab65330) (Abcam Cambridge, United Kingdom) following the manufacturer’s instructions. Briefly, after the samples were deproteinized, each sample was incubated with lactate assay buffer, lactate substrate mix, and lactate enzyme mix in a 96-well plate. After incubating for 30 minutes at room temperature, the optical density was measured at 450 nm with a microplate reader.

### Oxygen consumption

The O_2_ levels were measured using an Extracellular Oxygen Consumption Assay Kit (ab197243) (Abcam) following manufacturer’s protocol. The fluorescent dye used in this assay kit is quenched by oxygen and is excited at 360–380 nm (max 380) and emits at 630–680 nm (max 650), where the higher the fluorescence signal, the less oxygen is in the sample^78,79^. Cells were seeded in a 96-well plate under the same conditions described above, incubated with an O_2_ reagent, covered with mineral oil and read in a subsequent time course using a fluorometric microplate reader.

### Cytokines measurements

Enzyme-linked immune assays (ELISA) were performed to measure the levels of TNF-α, IL-6, IL-1β, and IL-10 in the culture supernatants following the manufacturer’s protocols (R&D® Systems, Inc., Minneapolis, MN, USA).

### Measurement of H_2_O_2_ levels

H_2_O_2_ quantification was performed after 24 hours, 48 hours and 7 days by an enzyme assay that uses 10-acetyl-3,7-dihydroxyphenoxazine to detect H_2_O_2_ following the manufacturer’s instructions (Amplex® Red Hydrogen Peroxide/Peroxidase Assay kit - Invitrogen®, Thermo Fisher Scientific). In a 96-well plate, the Amplex Red Reagents were mixed with the supernatant, incubated at room temperature for 30 minutes and then read with a microplate reader at 540 nm.

### Flow cytometry

Flow cytometry was performed to evaluate the percentage of F4/80+ macrophages (PE-conjugated anti-mouse F4/80 antibody clone T45-2342) (BD Biosciences Pharmingen, Franklin Lakes, New Jersey, USA) after the differentiation period. Cells were subsequently seeded in a 6-well plate, and the following reagents were used to stain and evaluate cell viability after 24 hours: PI (Thermo Fisher Scientific), a PE-conjugated anti-mouse TLR4 antibody (clone MTS510, BD Biosciences Pharmingen), an APC-Cy7-conjugated anti-mouse CD38 antibody (clone 90, Biolegend, San Diego, California, USA), an APC-conjugated anti-mouse CD206 antibody (clone C068C2, Biolegend, San Diego, California, USA), and a FITC-conjugated anti-mouse CD11b antibody (clone M1/70, BD Biosciences Pharmingen). Cells were acquired on a FACSCanto II flow cytometer (BD Biosciences Pharmingen) and analysed using FlowJo Software.

### Arginase activity

Arginase activity was measured as previously described^80^. BMDMs were cultured in a 6-well plate and incubated for 24 hours under the conditions described above. Briefly, the cells were lysed with 0.1% Triton X-100, after which a buffer containing 25 mM Tris-HCl and 5 mM MnCl_2_ was added and heated to activate the enzyme. Subsequently, the activated lysate was incubated with 0.5 M arginine to hydrolyse the arginine. The reaction was stopped by adding 400 μl of H_2_SO_4_:H_3_PO_4_:H_2_O. The urea level was measured at 540 nm after the lysates were incubated with 9% α-isonitrosophenone and heated at 100 °C for 45 minutes.

### NO measurement

We used the GRIESS reaction to measure the level NO in the supernatants of 7-day BMDMs cultures. Briefly, the day 7 supernatants were added to a 96-well plate and incubated with GRIESS reagents (1% sulfanilamide/0.1% N-(1-naphthyl) ethylenediamine dihydrochloride/2.5% H_3_PO_4_) for 15 minutes at room temperature and then read at 540 nm with a microplate reader.

### Western blot analysis

BMDMs were seeded in 6-well plates at a density of 2 × 10^6^ and incubated for 30 minutes. Cell lysates were generated using RIPA lysis buffer, and the protein extract was measured using a Pierce™ BCA Protein Assay kit (Waltham, Massachusetts, USA). To perform Western blotting, 20 µg of protein extract was separated in a polyacrylamide gel and then transferred to a nitrocellulose membrane using a semi-dry system. After transfer, the membranes were blocked using 5% non-fat dry milk in Tris-buffered saline containing Tween-20 (TBST) for 1 hour and then probed with a primary antibody overnight in a 5% BSA solution in TBST at 4 °C. The membranes were washed three times with TBST for 5 minutes each, probed with a secondary antibody for 1 hour and then washed three times with TBST for 5 minutes each. The membranes were developed using an Amersham Imager 680 blot and gel imager (Amersham, Buckinghamshire, United Kingdom).

Antibodies against mouse phospho-AKT, phospho-PI3k p85/p55, phospho-PKCα/βII, phospho-PKC-δ, phospho-p38, phospho-ERK1/2, phospho-SAPK/JNK, phospho-Ps6 and phospho-AMPKα (Cell Signaling Technology®, Danvers, Massachusetts, USA) were used as primary antibodies, and antibodies against mouse β-actin (Cell Signaling Technology®) or mouse GAPDH (Cell Signaling Technology®) were used as primary antibodies for normalization. For secondary antibodies, an HRP-conjugated goat anti-rabbit IgG H&L antibody (Abcam) was used. The relative densities of the bands were determined by densitometry analysis using Image Studio Lite Software Version 5.2 (LI-COR Biosciences, Lincoln, Nebraska, USA).

### Phagocytosis

First, we opsonized RBCs from sheep with an anti-sheep IgG antibody. Macrophages were plated onto a coverslip in a 12-well plate. After adhering, the cells were washed and cultured with the opsonized RBCs and the different glucose concentrations at a ratio of 30 targets to 1 macrophage. Non-opsonized RBCs were used as a control. After 60 minutes of incubation, the cells were washed, and the coverslips were coloured by Giemsa staining. The phagocytic index was calculated by counting 300 macrophages and determining how many had phagocytosed at least one opsonized RBC and the number of opsonized RBCs per phagocyte.

### Statistics

The results were evaluated by analysis of variance (ANOVA) followed by the Tukey-Kramer multiple comparisons test. The data are presented as the standard error of the mean (SEM), with p < 0.05 considered to represent a significant difference (GraphPad Prism 6). The tests were not conducted in a blind fashion.

## Supplementary information

the Supplementary Information
