# Supplementary material for: High Glucose Environments Interfere with Bone Marrow-Derived Macrophage Inflammatory Mediator Release, the TLR4 Pathway and Glucose Metabolism
Source: Sci Rep. 2019 Aug 7;9:11447. doi: 10.1038/s41598-019-47836-8 (PMC6686006; doi:10.1038/s41598-019-47836-8)

**High Glucose Environments Interfere with Bone Marrow-Derived Macrophage Inflammatory Mediator Release, the TLR4 Pathway and Glucose Metabolism**

Thais Soprani Ayala^1^, Fernando Henrique Galvão Tessaro^1^, Grasielle Pereira Jannuzzi^2^, Leonardo Mendes Bella^1^, Karen Spadari Ferreira^2^, Joilson O. Martins^1,*^

**Supplementary figure 1-** Western blot membranes probed with anti-mouse phosphor-AMPK, anti-mouse phosphor-S6 and anti-mouse β-actin.


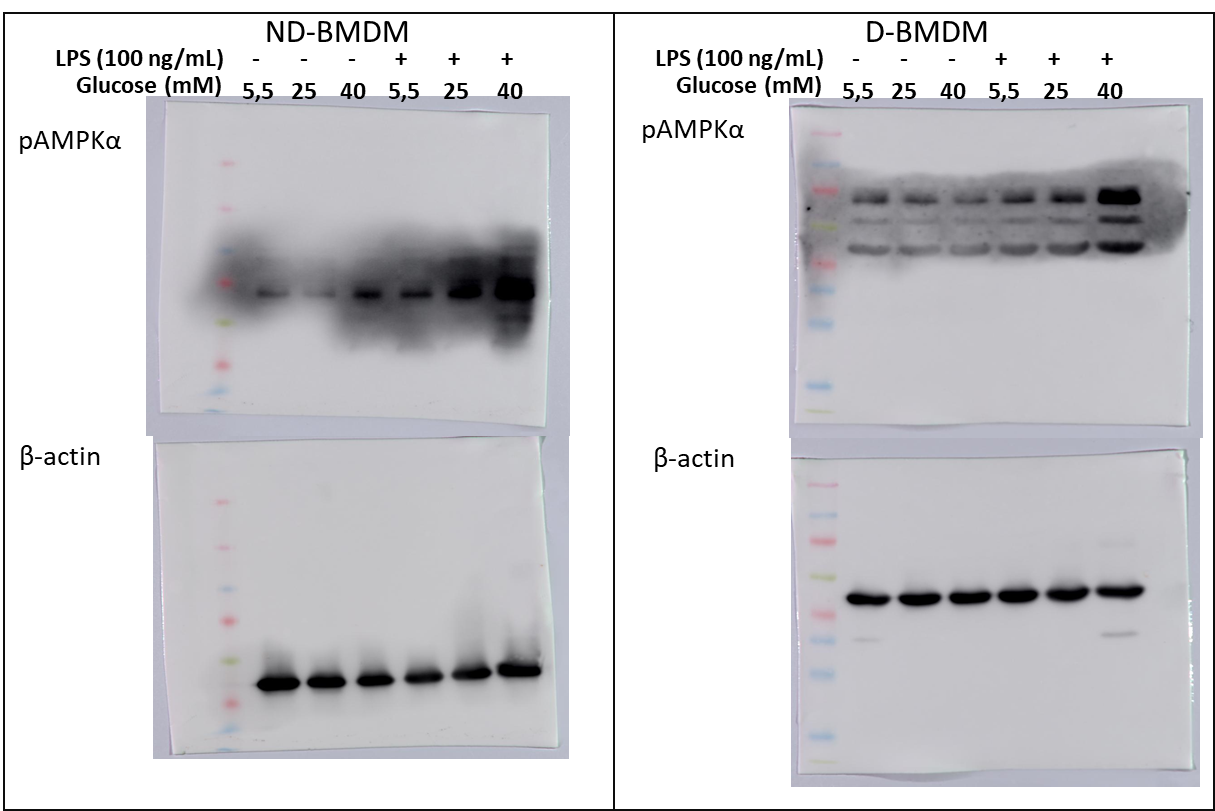


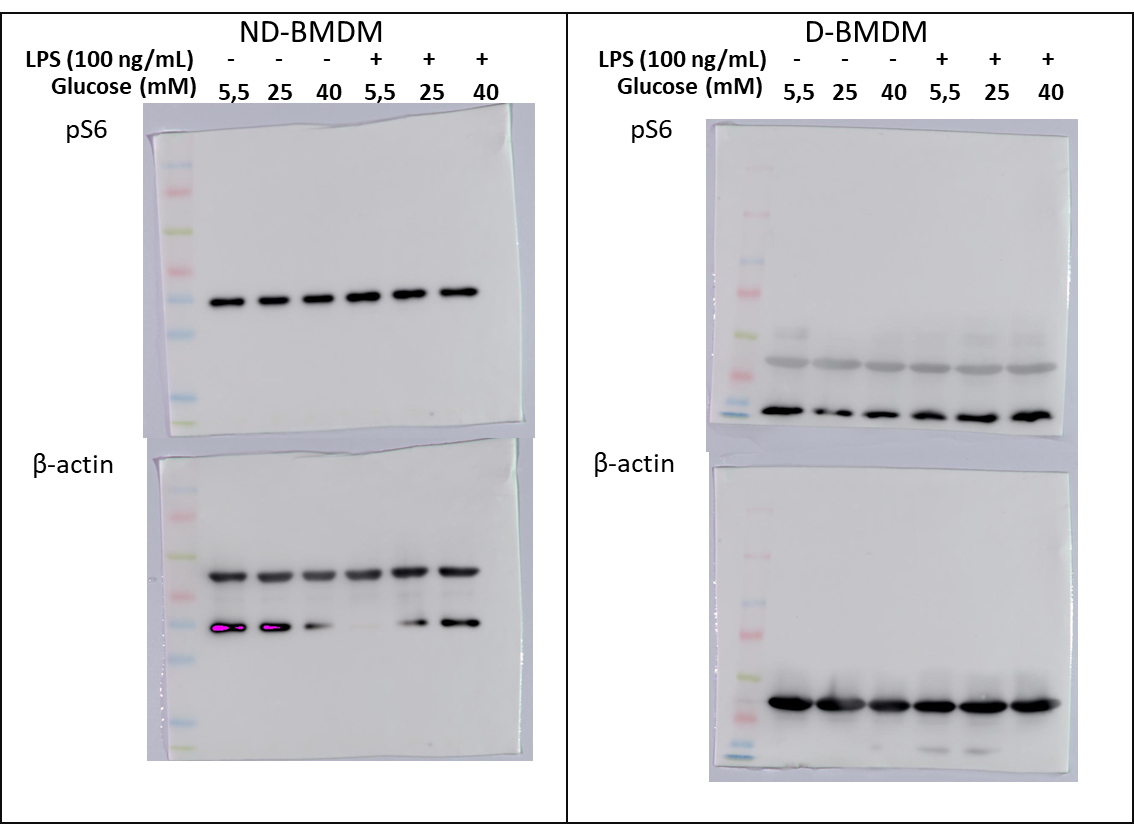


**Supplementary figure 2-** -Western blot membrane probed with anti-phospho-AKT, anti-phospho-PI3K p85/p55, anti-phospho-PKC-α/βII, anti-phospho-PKC-δ, anti-mouse β-actin, and anti-mouse GAPDH.


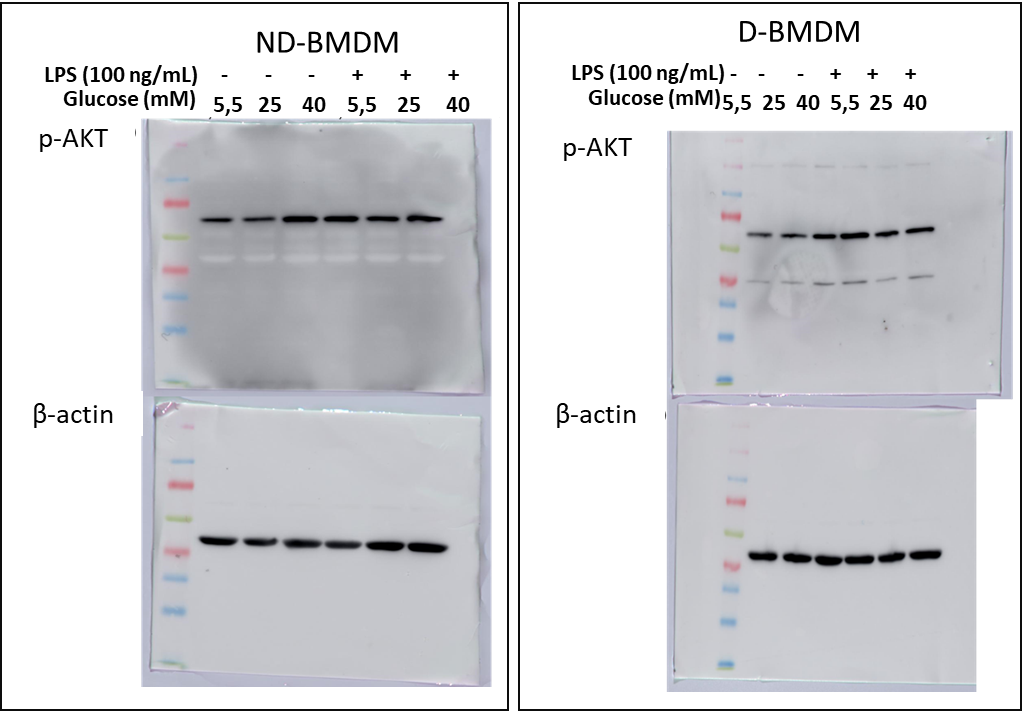


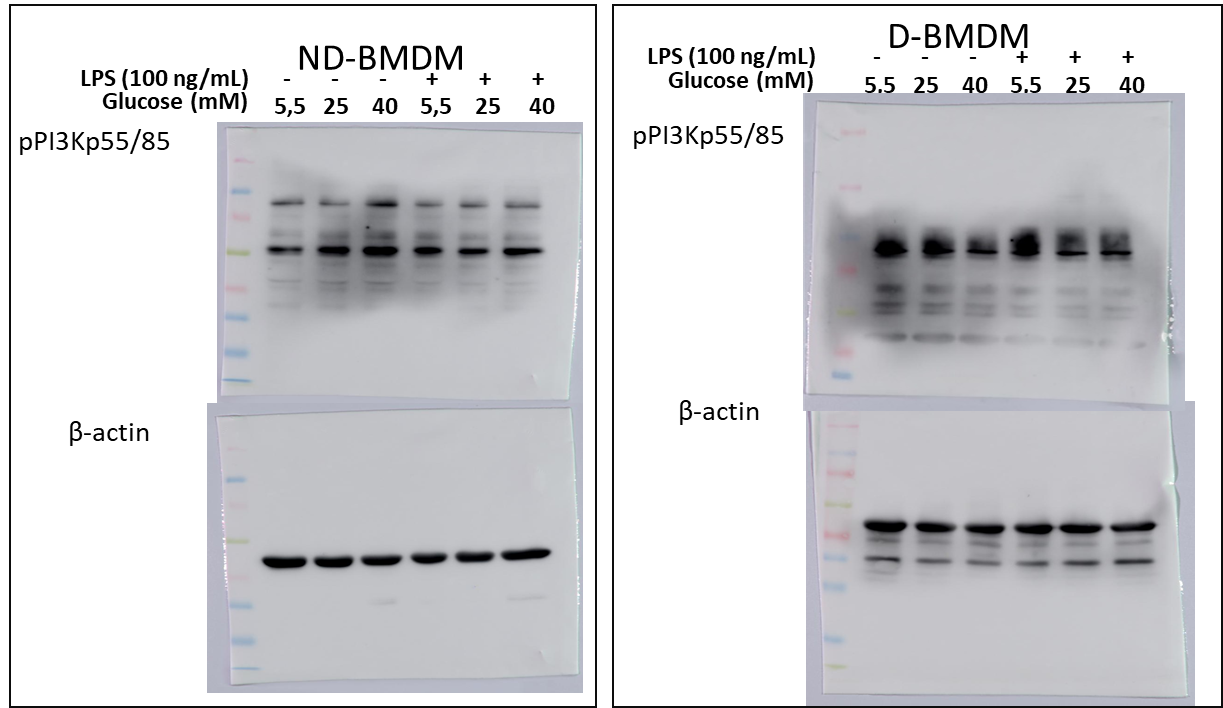


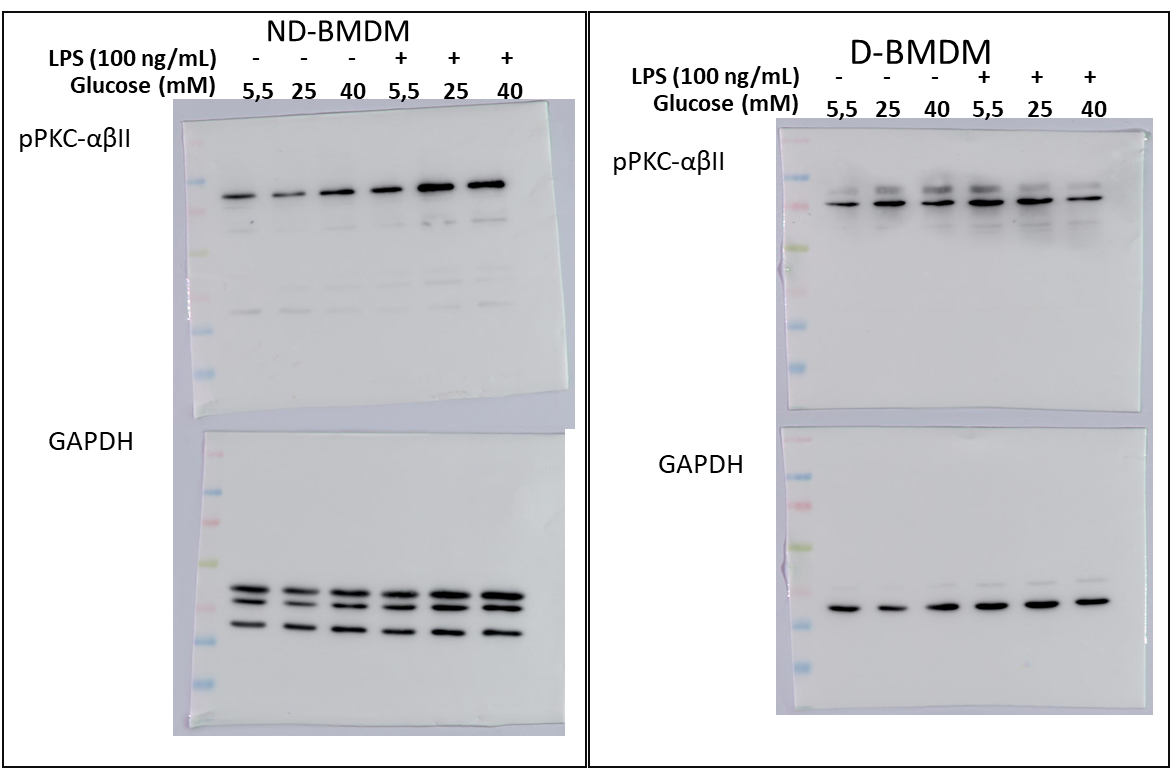


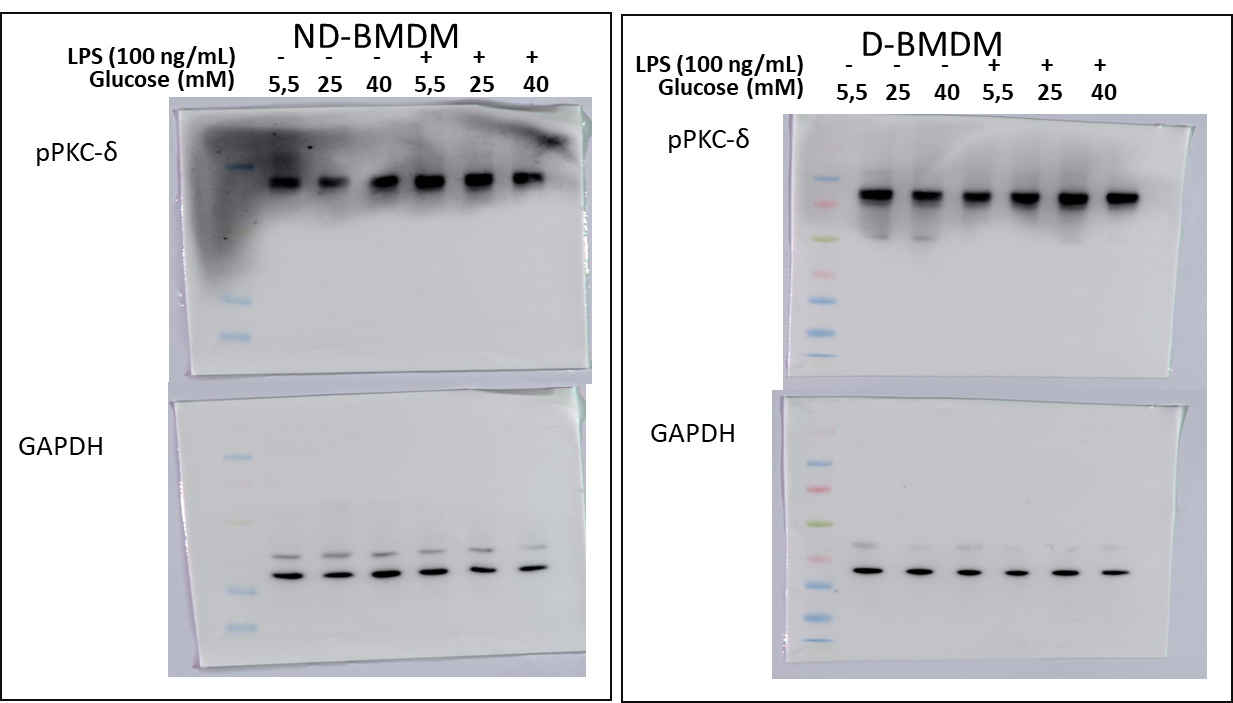


Supplementary figure 3- Western blot membrane probed with anti-phospho-SAPK/JNK, anti-phospho- ERK 1/2, anti-phospho-P38 and anti-GAPDH.


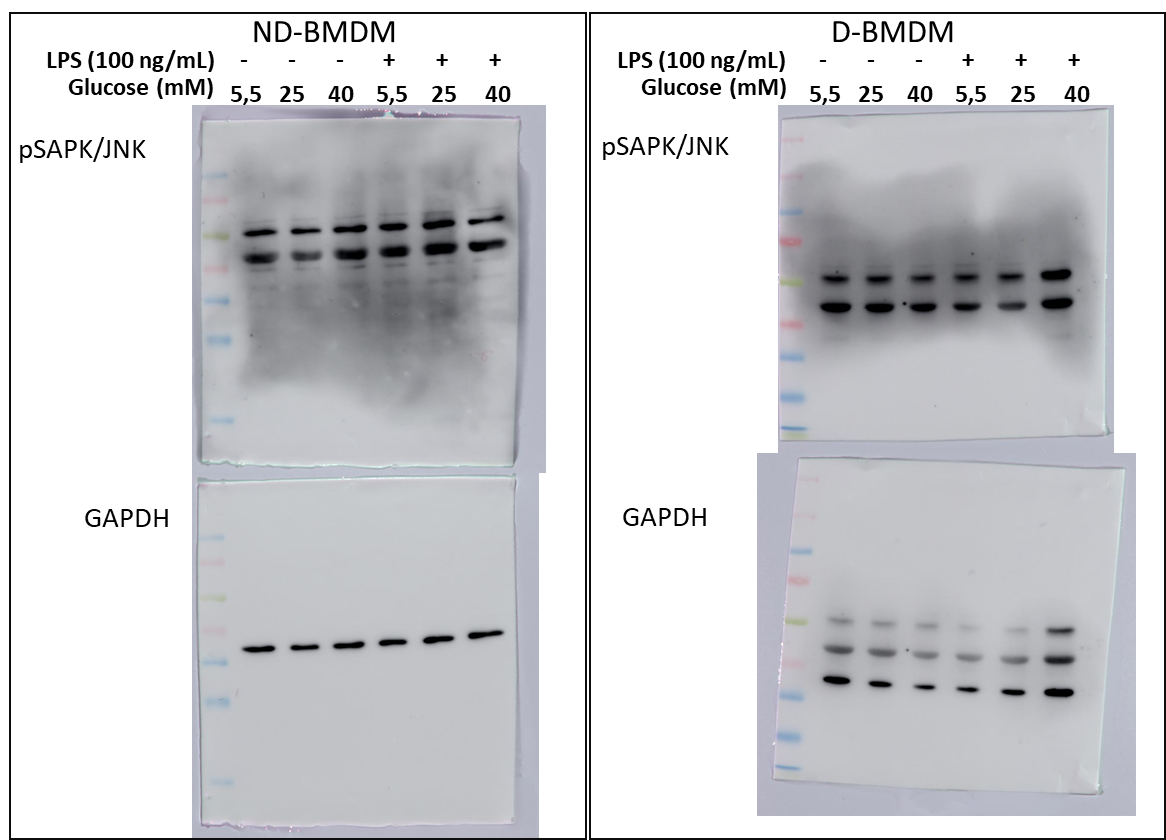


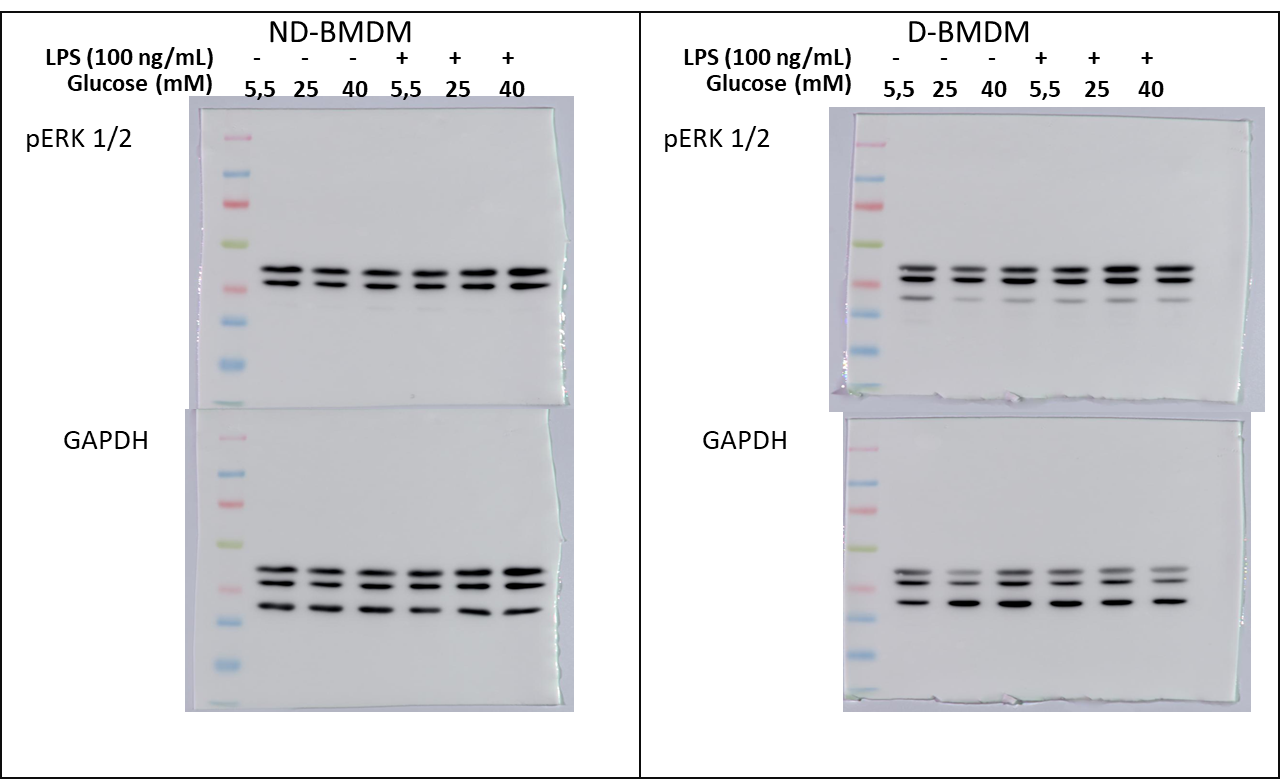


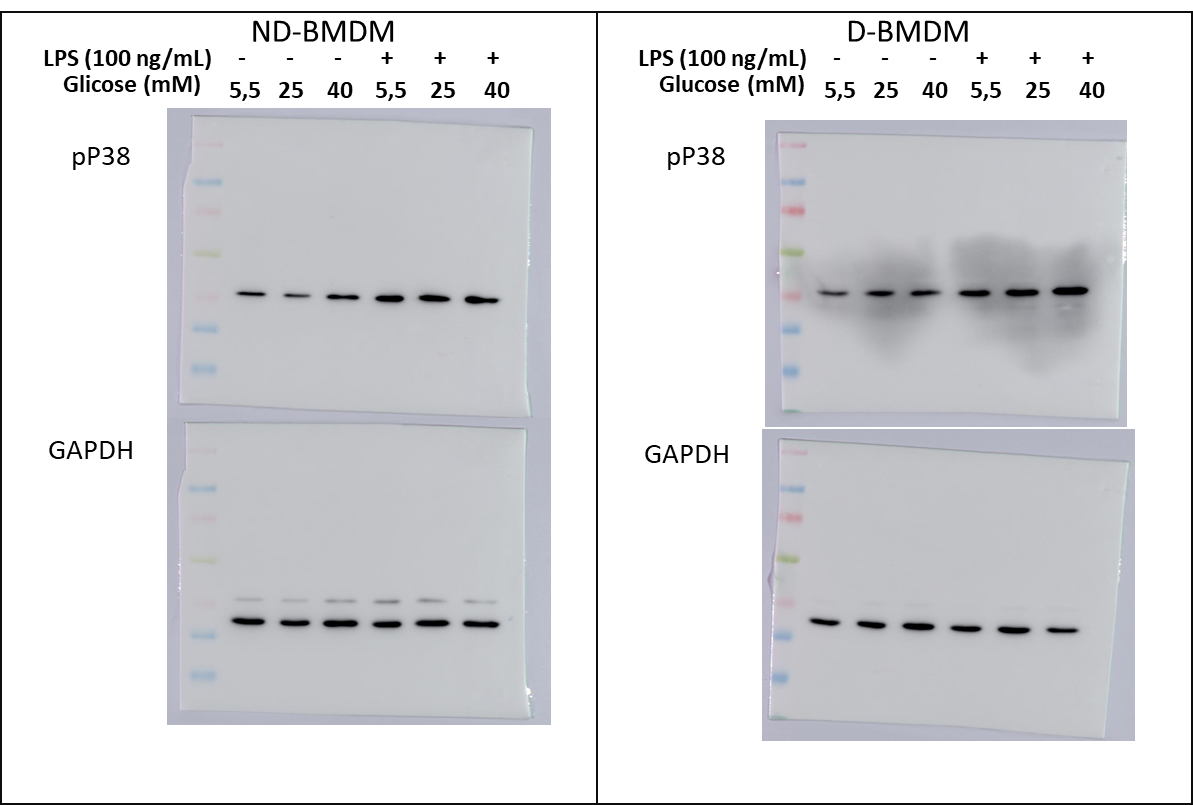

Supplement: Supplementary file 1 — the Supplementary Information [file 41598_2019_47836_MOESM1_ESM.docx]
